# Editorial: Management of arrhythmias in acute and intensive care settings

**DOI:** 10.3389/fcvm.2026.2020556

**Published:** 2026-10-27

**Authors:** Martin Balik, Stepan Havranek

**Affiliations:** 1 Department of Anesthesiology and Intensive Care, 1st Faculty of Medicine, Charles University and General University Hospital in Prague, Prague, Czechia; 2 2nd Department of Medicine – Department of Cardiovascular Medicine, 1st Faculty of Medicine, Charles University and General University Hospital in Prague, Prague, Czechia

**Keywords:** amiodarone, atrial fibrillation, catheter ablation, ganglion stellate block, intensive care, propafenone, ventricular arrhyhtmias

Cardiac arrhythmias become frequent in general population which transmits to the incidence among the critically ill (1). As an independent mortality predictor, they represent either a primary problem or consequence of the underlying critical illness and an important driver of further hemodynamic deterioration (2, 3). The evidence in critical care arrhythmia management is limited because some of the conclusions generated on large cohorts of cardiology outpatients cannot be applied in the acute and intensive care setting. Hence, the decisions are often driven by available bedside imaging and applied physiology (4).

Shortened diastolic filling, loss of atrial contribution, irregular ventricular activation, increased myocardial oxygen demand, reduced coronary perfusion, and impaired ventricular function may all contribute to a rapid decline in cardiac output, particularly in patients with pre-existing ventricular dysfunction or limited cardiovascular reserve (5, 6). The resulting hypotension and congestion, sympathetic activation and myocardial ischemia in turn facilitate recurrent arrhythmia. Thus, a self-perpetuating cycle may develop in which arrhythmia aggravates hemodynamic compromise, while hemodynamic and metabolic deterioration further increases arrhythmogenicity (5–7).

Potentially reversible precipitants—including acute myocardial ischemia, electrolyte abnormalities, acidosis, hypoxemia, elevated filling pressure, infection, drug toxicity, and proarrhythmic medication—must be identified and corrected simultaneously (7–9). Causality of arrhythmias requires meticulous clinical assessment including the extra-coronary vascular lesions in recurrent ischemia despite patent grafts. The left subclavian artery stenosis was identified as the cause for aVR ST-elevation by limiting LIMA graft inflow in post-CABG patient by Li et al.

Bedside echocardiography plays a key role in assessment of the ventricular systolic function, filling pressures, atrial size/function and prediction of rhythm control, valve disorders and is helpful in decision on adequate anticoagulation (10). The complex assessment should enable more efficient pharmacological treatment avoiding cardiac depressant medication in already compromised contractility, on the other hand, a universal application of amiodarone associates with less efficacy, worse outcome data in patients with non-dilated left atrium and well known multiorgan toxic side effects (11–13).

The state-of-the-art review by Tencer et al. offers a step-by-step approach including correction of triggers and individualized selection of a rate- vs. rhythm-control strategy based on echocardiography. Blending physiology observations with existing randomized trials allows for refinement of the therapeutic strategies in this high-risk population. The exploratory study by Balik et al. of the trial on supraventricular arrhythmias (SVA) in mechanically ventilated patients with septic shock (NCT03029169) (14) demonstrated that in presence of right ventricular (RV) dysfunction, propafenone was associated with four to five times fewer arrhythmia recurrences compared with amiodarone, despite similar short-term cardioversion rates. The RV dysfunction diagnosed by echocardiography included patients with decompensated cor pulmonale challenging a vague definition of structural heart disease often presented as alleged contraindication to the 1C class antiarrhythmics. Under certain conditions (Figure 1) the propafenone should be preferred to amiodarone for rhythm control in patients with RV dysfunction.

**Figure 1:**
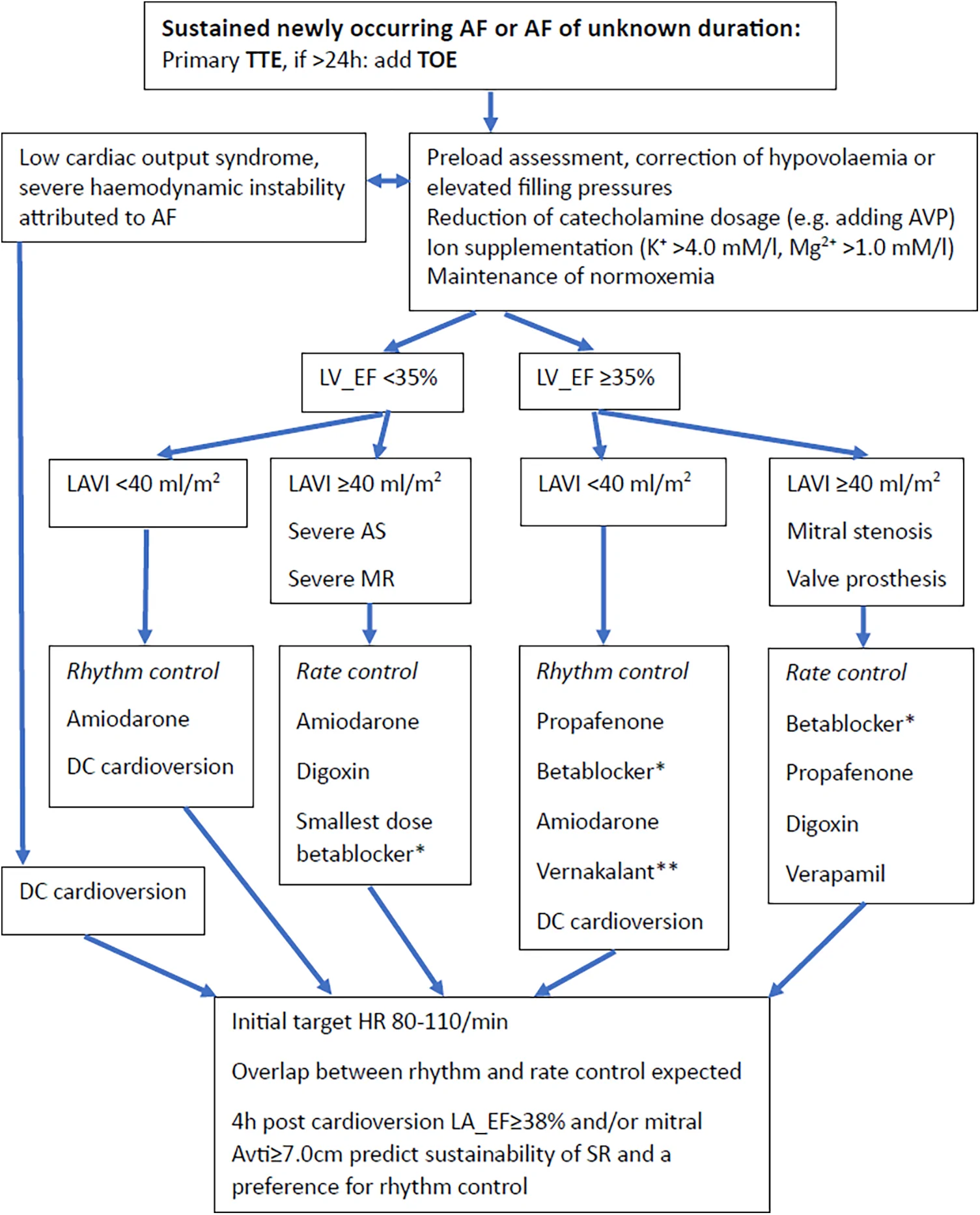
Algorithm for management of NOAF or AF of unknown duration (10). Current evidence prefers a rhythm control for all with non-dilated left atrium (i.e., LAVI<40 mL/m^2^). In addition, presence of a right ventricular compromise indicates propafenone as the agent of choice. Indicated lists in the boxes on pharmacotherapy start with the most preferred agents. *At the initial phase in the ICU the short-acting betablockers like landiolol or esmolol are recommended. **Vernakalant may be a primary treatment if available.

The potential role of an autonomic modulation by the stellate ganglion blockade (SGB) in refractory arrhythmias was explored in the narrative review by Zhang and An. Additionally, the SGB influences stress responses, perception of pain and multiple postoperative outcomes among surgical patients. SGB also produced vagus nerve stimulation effects, encompassing insomnia, depression, and cognitive impairment.

In patients with severe hemodynamic instability, temporary mechanical circulatory support, such as VA-ECMO or Impella, may serve as a bridge to successful treatment, including catheter ablation (CA). The most established role for urgent CA is in patients with incessant monomorphic ventricular tachycardia (VT) and electrical storm. Ablation may also be considered when recurrent polymorphic VT or ventricular fibrillation (VF) is initiated by a reproducible premature ventricular complex, including Purkinje-related triggers. Both standard radiofrequency and novel pulsed field ablation have acceptable results in reducing VT episodes (7, 8, 15). CA may be feasible in incessant recurrent paroxysmal supraventricular tachycardia, atrial flutter or pre-excited atrial arrhythmias. When refractory AF with high ventricular rate causes hypotension and circulatory compromise, AV node ablation and physiological pacing could be an option (16). Recent data support concept pace and ablate with promising results on left ventricular function and morphology of QRS complex (17).

CA require multiple imaging modalities including fluoroscopy, 3D electroanatomical mapping and intracardiac echocardiography. Zero-fluoroscopy approach is current trend in many centres. The study by Stec et al. demonstrated safe and effective ablation of maternal supraventricular tachycardia and idiopathic ventricular arrhythmias during pregnancy without fluoroscopy including the 5-year follow up. The complex approach underlines the importance of pregnancy heart teams.

Patients with pulmonary hypertension (PH) can be sensitive to a loss of rhythm control (18). SVA in PH patients was associated with severe symptoms and increased mortality, and restoration of sinus rhythm improved clinical outcomes (19, 20). An exploratory study by Dusik et al. of the randomized trial (NCT04053361) unveiled that an extensive CA for AF and atrial tachycardia in patients with PH did not reduce arrhythmia recurrence during more than 40 months follow-up. Nonetheless, the cavo-tricuspid isthmus ablation appeared effective in preventing recurrence of typical atrial flutter.

The role of biomarkers in heart failure diagnosis lays also in predicting a response to treatment. In patients with AF, data are inconclusive, especially for the rhythm control strategy. The study by Stekla et al. showed that growth differentiation factor 15 (GDF-15) level changes after radiofrequency and pulsed field CA of AF. This protein from the transforming growth factor β (TGF-β) cytokine family is a potent marker of bleeding adverse events in anticoagulated patients with AF and a predictor of overall mortality.

A high-risk profile of a patient with AF does not mean anticoagulation is contraindicated. The narrative review by Kim et al. suggested that proton pump inhibitor co-therapy in patients receiving non-vitamin K antagonist oral anticoagulants may represent a pragmatic strategy.

The case series by Elahi et al. gives insight on the temporary use of theophylline in patients with sinus pauses. Risk of associated AF warrants further research.

In a study by Wu et al., deep learning-based early prediction of life-threatening ventricular arrhythmias using long-term Holter ECG signals was shown as efficient compared with the traditional models and offers an extensible solution in real-time with a high degree of accuracy.

## Conclusion

Management of arrhythmias encompasses careful identification and correction of reversible triggers, bedside imaging, and tailored selection of antiarrhythmic, anticoagulation, autonomic-modulating strategies, circulatory support, and CA. Advances in precise diagnostics, CA methods (including navigational techniques), biomarker understanding, and artificial intelligence applications may further expand the options available in this challenging population.
